# A Novel Delivery System for the Controlled Release~of Antimicrobial Peptides: Citropin 1.1 and Temporin A

**DOI:** 10.3390/polym10050489

**Published:** 2018-05-02

**Authors:** Urszula Piotrowska, Ewa Oledzka, Anna Zgadzaj, Marta Bauer, Marcin Sobczak

**Affiliations:** 1 Department of Biomaterials Chemistry, Chair of Inorganic and Analytical Chemistry, Faculty of Pharmacy with the Laboratory Medicine Division, Medical University of Warsaw, Banacha 1 St., 02-097 Warsaw, Poland; eoledzka@wum.edu.pl (E.O.); marcin.sobczak@e-mail.com (M.S.); 2 Department of Organic Chemistry and Biochemistry, Faculty of Materials Science and Design, Kazimierz Pulaski University of Technology and Humanities in Radom, 27 Chrobrego St., 26-600 Radom, Poland; 3 Department of Environmental Health Science, Faculty of Pharmacy with the Laboratory Medicine Division, Medical University of Warsaw, 1 Banacha St., 02-097 Warsaw, Poland; azgadzaj@wum.edu.pl; 4 Department of Inorganic Chemistry, Faculty of Pharmacy with the Laboratory Medicine Division, Medical University of Gdansk, Al. Gen. J. Hallera 107 St., 80-416 Gdansk, Poland; bauerm@gumed.edu.pl

**Keywords:** antimicrobial peptides, biodegradable polymers, biocompatible polymers, drug delivery systems, controlled release, citropin, temporin, ionic liquids

## Abstract

Antimicrobial peptides (AMPs) are prospective therapeutic options for treating multiple-strain infections. However, clinical and commercial development of AMPs has some limitations due to their limited stability, low bioavailability, and potential hemotoxicity. The purpose of this study was to develop new polymeric carriers as highly controlled release devices for amphibian peptides citropin 1.1 (CIT) and temporin A (TEMP). The release rate of the active pharmaceutical ingredients (APIs) was strongly dependent on the API characteristics and the matrix microstructure. In the current work, we investigated the effect of the polymer microstructure on in vitro release kinetics of AMPs. Non-contact laser profilometry, scanning electron microscopy (SEM), and differential scanning calorimetry (DSC) were used to determine the structural changes during matrix degradation. Moreover, geno- and cytotoxicity of the synthesized new carriers were evaluated. The in vitro release study of AMPs from the obtained non-toxic matrices shows that peptides were released with near-zero-order kinetics. The peptide “burst release” effect was not observed. New devices have reached the therapeutic concentration of AMPs within 24 h and maintained it for 28 days. Hence, our results suggest that these polymeric devices could be potentially used as therapeutic options for the treatment of local infections.

## 1. Introduction

Our life expectancy has increased as the quality of medical care is constantly improving and new therapies are being developed all the time. However, microorganism resistance to antibiotics has become an important challenge in modern medicine due to the global uncontrolled use of antibiotics [1]. After the golden age of antibiotic discovery, only two new classes of antibiotics have been marketed [2,3].

Antimicrobial peptides (AMPs), also called host defense peptides or cationic peptide antibiotics, are a prospective therapeutic option for treating multiple-strain infections. AMPs represent a diverse class of naturally occurring biologically active molecules with a broad spectrum of activity. They possess activity against viruses, both Gram-positive and Gram-negative bacteria (with endotoxin-neutralizing activity), fungi, and protozoa. Moreover, AMPs display anti-inflammatory, immunomodulatory, antitumor, angiogenic, and wound healing properties [4,5,6].

However, from the therapeutic point of view, clinical and commercial development of AMPs as low-molecular-weight active pharmaceutical ingredients (APIs) still has some limitations. AMPs have limited stability, low bioavailability, and potential hemotoxicity. In order to overcome the above-mentioned issues, peptides could be loaded into polymeric drug delivery systems (DDSs). Non-toxic polymeric matrices with appropriate microstructure would be able to release APIs with optimal pharmacokinetics, improving the efficacy and toxicological safety of the therapy [7]. Novel DDSs, which are characterized by highly controlled drug release profiles, are currently being demanded by both the pharmaceutical industry and medical practitioners.

Recent studies focused on the development of polymeric carriers for AMPs or short lipopeptides. The tested matrices were poly (lactic acid-co-castor oil) [8], poly (ester-anhydride) [8], poly(ε-caprolactone) (PCL) [9], copolymer of ε-caprolactone and trimethylene carbonate [9] and polyphosphoesters [10]. New systems were reported to be effective against *Enterococcus faecalis* [8], *Bacillus anthracis* [9], *Enterococcus hirae* [9], *Staphylococcus aureus* [9,10], *Escherichia coli* [10] and *Pseudomonas aeruginosa* [10]. However, the previous studies did not take into account the determination of the kinetics and mechanisms of the peptide release from the new systems. Moreover, there are no studies based on released amount of peptides with reference to therapeutic index (TI). AMPs due to their high hydrophobicity are able to interact not only with bacterial cell membranes, but also with red blood cells, so they show hemolytic activity in certain concentrations. Therefore, it is very important to achieve a therapeutic concentration of peptides in the target tissues. The TI for AMPs defined by Chen et al. [11] is a ratio that compares the minimal hemolytic concentration (MHC) that induces erythrocyte lysis and the minimal inhibitory concentration (MIC) that inhibits the visible growth of the bacterium being investigated. Larger TI values indicate simultaneously greater antimicrobial specificity of the peptide molecule.

In this study, new polymeric matrices were synthesized by enzymatic ring opening polymerization (eROP) of ε-caprolactone (CL) in order to obtain non-toxic devices for highly controlled and prolonged release of amphibian AMPs: citropin 1.1 (CIT) and temporin A (TEMP).

CIT, a 16–amino acid (GLFDVIKKVASVIGGL-NH_2_) cationic peptide (+2 at pH 7) is produced by the tree frog *Litoria citropa* [12]. It has a high content of hydrophobic residues (56%) in its molecule and an average molecular mass of 1397 Da. CIT is one of the simplest amphibian peptides with a broad spectrum of biological activity reported to date. The solution structure of CIT is an alpha helix with well-defined hydrophobic/hydrophilic regions. CIT has shown antimicrobial activity toward Gram-positive bacteria (*Staphylococcus aureus*, including methicillin-resistant *Staphylococcus aureus*, *Rhodococcus equi*, *Bacillus subtilis*) with antibiofilm properties [13,14]. Moreover, it has antifungal and antitumor activity [15,16].

TEMP is a short, 13–amino acid peptide (FLPLIGRVLSGIL-NH_2_) isolated from skin secretions of the European red frog *Rana temporaria*. It has a positive net charge (+2 at pH 7) and a higher content of hydrophobic residues (61%) than CIT. Average molecular mass of TEMP is 1615 Da. It has broad antimicrobial activity against both Gram-positive and Gram-negative bacteria [17]. We chose ionic liquids (ILs) as an alternative to the volatile organic solvents during poly(ε-caprolactone) PCL synthesis [18]. ILs are commonly known as solvents that enhance enzyme stability and activity. Moreover, they affect the microstructure of the polymers [19]. The amount of drug released was mainly dependent on the kind of polyester, its number average molecular weight (*M_n_*), dispersity (*M_w_*/*M_n_*), or microstructure. However, our main intention was to check how the kinetics of peptide release might depend on the microstructural characteristics of the polyester matrices.

## 2. Materials and Methods

### 2.1. Materials

CL (≥97%; Aldrich, Poznan, Poland) was dried and distilled over CaH_2_ at reduced pressure before use. Immobilized lipase B from *Candida antarctica* (CALB) (catalog #L4777) was purchased from Sigma, Poland. 1-Butyl-3-methylimidazolium bis(trifluoromethylsulfonyl)imide ([bmim][NTf_2_] ≥98%; Aldrich, Poznan, Poland) and 1-butyl-3-methylimidazolium hexafluorophosphate ([bmim][PF_6_] ≥ 98%, Merck, Poland) were used as received. CIT (≥95%) and TEMP (≥95%) (Lipopharm.pl, Poland) were used as received. Dichloromethane (DCM), toluene, and methanol (Avantor, Gliwice, Poland) were used as received. Phosphate buffered saline (PBS) was purchased from Sigma (Poznan, Poland) and was also used as received. Dulbecco’s modified Eagle’s medium (DMEM) was purchased from Gibco (Thermo Fisher Scientific, Waltham, MA, USA).

### 2.2. Synthesis of Polymeric Matrices

In a typical experiment, the substrate (3.0 g of CL) was mixed with solvent (3.0 mL) under argon atmosphere, followed by the addition of 300 mg CALB, according to our previously described method [20]. Finally, the obtained PCL was precipitated in cold methanol, filtered, and dried under vacuum at room temperature for 4 days.

### 2.3. Spectroscopy Data of PCL

^1^H-NMR (CDCl_3_, δ, ppm): 4.07 [2H, t, –CH_2_CH_2_CH_2_CH_2_C**H_2_**OC(O)–], 3.66 [2H, t, –C**H_2_**OH], 2.32 [2H, t, –CH_2_CH_2_CH_2_CH_2_C**H_2_**COO–], 1.67 [4H, m, –CH_2_C**H_2_**CH_2_C**H_2_**CH_2_COO–], 1.39 [2H, m, –CH_2_CH_2_C**H_2_**CH_2_CH_2_COO–]; ^13^C-NMR (CDCl_3_, δ, ppm): 173.7 [–**C**(O)O–], 64.3 [–CH_2_CH_2_CH_2_CH_2_**C**H_2_OC(O)–], 62.7 [–**C**H_2_OH] 34.3 [–CH_2_CH_2_CH_2_CH_2_**C**H_2_COO–], 28.5 [–CH_2_CH_2_CH_2_**C**H_2_CH_2_OC(O)–], 25.7 [–CH_2_CH_2_CH_2_**C**H_2_CH_2_COO–], 24.7 [–CH_2_CH_2_**C**H_2_CH_2_CH_2_COO–]; FT-IR (KBr, cm^−1^): 3440 (νO-H), 2938 (ν_as_CH_2_), 2866 (ν_as_CH_3_), 1727 (νC=O), 1245 (νC-O).

### 2.4. Preparation of Poly(ε-Caprolactone) Devices of CIT and TEMP

The obtained PCL matrices were dissolved in DCM under argon atmosphere. Then, the CIT or TEMP was slowly added to the vigorously stirred PCL solution. The samples were dried in vacuo at room temperature until a constant weight was reached. The tablet disc (13 mm diameter, 1 mm thick) was obtained using a hydraulic press (Specac, London, UK) at 98 kN. The mean weight of the developed devices was about 200 mg, corresponding to approximately 5 mg of peptide.

### 2.5. Genotoxicity Assay of Polymeric Matrices

The *umu*-test was performed with *Salmonella typhimurium* TA1535/pSK1002 in 96-well microplates according to the ISO 13829 protocol with and without metabolic activation, as we described before [21].

### 2.6. Neutral Red Uptake (NRU) Assay of Polymeric Matrices

Cytotoxicity assays were performed on BALB/c 3T3, clone A31 mammalian fibroblasts (American Type Culture Collection) on the basis of the ISO 10993-5:2009 guideline: Biological evaluation of medical devices—Part 5: Tests for in vitro cytotoxicity. Cells were seeded into 96-well microplates (10^4^ cells·mL^−1^) in DMEM culture medium (supplemented with 10% calf bovine serum, 100 IU/mL penicillin, and 0.1 mg·mL^−1^ streptomycin) and incubated for 24 h (5% CO_2_, 37 °C, >90% humidity). Subsequently wells were examined under a microscope, and culture medium was replaced with the tested extracts (prepared as for the *umu*-test) mixed with the treatment medium (1:1). All extracts were tested in twofold dilution series for 24 h (4 data points for each one). The next day, cells were washed with PBS and treated with the neutral red medium for 3 h and then with desorbing fixative (ethanol and acetic acid water solution). The amount of neutral red accumulated by cells was evaluated colorimetrically at 540 nm. PBS and SLS were used as negative and positive controls, respectively. The cell viability was calculated relative to the negative control.

### 2.7. Tests for In Vitro Cytotoxicity of Polymeric Matrices

For the cytotoxicity test, agar cells were seeded (1.5 × 10^5^ cells·mL^−1^, DMEM supplemented as for the NRU test) into 6-well plates and maintained in culture for 24 h. The next day, each plate was examined under a microscope, and the culture medium was replaced with fresh DMEM with 1% agar. Round or square sterile samples (about 1 cm diameter/side length) of all tested materials were placed carefully on the solidified agar layer in each well. As negative and positive controls, polyethylene foil and latex were used, respectively. In each test one well seeded with cells was left with clear surface as a control of the cell culture. After 24 h, cells were examined under a microscope to determine the cytotoxic effect before and after carefully removing the specimens from the agar.

### 2.8. In Vitro Studies of Citropin 1.1 and Temporin A Release from Polymeric Devices

The polymeric devices were immersed in 0.1 M PBS (10 mL) at 37 °C and gently shaken. The sample solutions were withdrawn for analysis at selected time intervals and replaced with new buffer solution. The quantity of peptide was analyzed by high-performance liquid chromatography (HPLC). The samples were prepared in triplicate.

### 2.9. Measurements

The polymerization products were characterized by means of ^1^H and ^13^C NMR (Varian 300 MHz, Palo Alto, CA, USA) at room temperature, with CDCl_3_ as solvent. Fourier transform infrared (FT-IR) spectra were measured from KBr pellets (Perkin Elmer spectrometer, Perkin Elmer, Warsaw, Poland).

SEC traces were recorded using an Agilent 1100 Series isocratic pump, a degasser, an autosampler thermostatic box for columns, and a set of TSKgel columns (2 × PLGel 5 microns MIXED-C) at 30 °C. A Wyatt Optilab rEX interferometric refractometer and a MALS DAWN EOS laser photometer (Wyatt Technology Corp., Santa Barbara, CA, USA) were used as detectors. Methylene chloride was used as the eluent, at a flow rate of 0.8 mL·min^−1^. *M*_n_ and *M*_w_/*M*_n_ were calculated from the experimental traces using the Wyatt ASTRA v 4.90.07 program.

*M*_n_ and *M*_w_*/M*_n_ of the obtained polyesters were determined by SEC/MALS with a system composed of an Agilent 1100 chromatograph series with an isocratic pump, an autosampler, a degasser, and a thermostatic box for columns. Refractive index (OPTILAB rex, Wyatt Technology Corporation, Santa Barbara, CA, USA) and MALS (DAWN EOS, Wyatt Technology Corporation, Santa Barbara, CA, USA) were applied as detectors and the TSKgel HXL columns were used in series for separations. DCM was used as eluent at a flow rate of 0.8 mL·min^−1^.

The matrix-assisted laser desorption/ionization mass spectrometry (MALDI-ToF-MS) experiments were performed on an Axima-Performance TOF spectrometer (Shimadzu Biotech, Manchester, UK), equipped with a nitrogen laser (337 nm) using a dithranol (1,8-dihydroxy-9(10H)-anthracenone) matrix.

Thermogravimetric analyses (TGA) were performed using TA Instruments Q50 (New Castle, DE, USA) at a heating rate of 10 °C·min^−1^ under nitrogen flow (60 mL∙min^−1^). Differential scanning calorimetry (DSC) measurements were performed using a TA Instruments DSC Q20 (New Castle, DE, USA) with aluminum pans at a heating rate of 10 °C min^−1^ under nitrogen flow (50 mL∙min^−1^). The degree of crystallinity (*X_c_*) was calculated from the peak enthalpies normalized to the actual weight fraction of polymer using Equation (1):

*X_c_* = [Δ*H_f_* (*T_m_*)] × [Δ*H_f_*_0_ (*T*_0*m*_)]^−1^ (1)

where Δ*H_f_* (*T_m_*) is the enthalpy of fusion at the melting point and Δ*H_f_*_0_(*T*_0*m*_) is the heat of fusion of 100% crystalline PCL (139.5 J∙g^−1^).

Morphological assessment of the samples was carried out with a FEI Quanta 250 FEG scanning electron microscope (SEM) (FEI Inc., Eindhoven, The Netherlands).

Surfaces of tablet matrices were determined with the help of a Contact 3D surface profiler (Taylor Hobson TalySurf CCI Lite, Ametek, UK).

HPLC was performed using a Waters apparatus with the use of an Agilent ZORBAX column (1.8 um, SB-C18, 4.6 × 50 mm). The mobile phase consisted of water (0.1% formic acid) and acetonitryl (0.1% formic acid) at a flow rate of 1 ml·min^−1^. 

### 2.10. Mathematical Models for Peptide Release Studies

The release data points were subjected to zero-order and first-order kinetics and Higuchi and Korsmeyer–Peppas models [22,23,24].

Zero-order model:

(2) F=kt

First-order model:

(3) $$\log F=\log F_{0}-\frac{kt}{2.303}$$

Korsmeyer–Peppas model:

(4) $$F=kt^{n}\;(F<0.6)$$

where *F* is the fraction of peptide released up to time (*t*), *F*_0_ is the initial concentration of peptide, *k* is the constant of the mathematical models, and *n* is the exponent of the Korsmeyer–Peppas model.

## 3. Results and Discussion

### 3.1. Characterization of PCL Matrices

The purpose of our work was to obtain nontoxic PCLs as devices for highly controlled release of CIT and TEMP. Biodegradable polyesters were synthesized using CALB as a biocatalyst. Lipase was used to avoid the difficulty of completely removing the residual of the conventional metal catalyst from the final product. This paper is a continuation of our earlier investigations [20]. The polymerization was carried out at 60 and 80 °C for 7 days in two ILs: [bmim][NTf_2_] and [bmim][PF_6_]. Moreover, the process was performed without solvent to compare the effect of ILs on PCL characterization.

As is commonly known, many factors can influence the release rate of API from polymeric matrices. It is mainly dependent on the kind of polymer and its *M*_n_, *M*_w_/*M*_n_, and microstructure. However, our main intention was to check how peptide release kinetics could be dependent on the polymeric matrix microstructure, thus polyester matrices were synthesized and characterized (Table 1).

**Figure 1:**
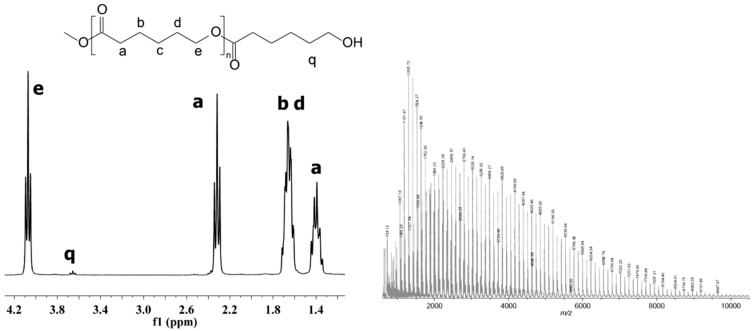
^1^H NMR and MALDI-ToF mass spectrum of synthesized PCL-1 analyzed in CDCl_3_. The reaction was carried out using CALB and [bmim][NTf_2_] as solvent at 80 °C for 7 days.

The chemical structure of the obtained homopolymers was confirmed by ^13^C, ^1^H NMR (Figure 1) and IR studies (Experimental section).

The *M*_n_ of PCLs synthesized in ILs was determined by SEC and was in the range of 2100–6100 Da. The *M*_w_*/M*_n_ indices were about 1.5 for homopolymers synthesized in [bmim][PF_6_] and about 1.9 for homopolymers synthesized in [bmim][NTf_2_]. The reaction yields ranged from 78 to 88%. The results obtained in bulk conditions confirm our earlier assumptions that ILs improve enzyme stability at higher temperatures and lead to obtaining polyesters with higher *M_n_*, lower *M_w_/M_n_*, and a higher conversion.

The thermal properties of the synthesized matrices determined by the DSC method showed that PCLs synthesized in [bmim][NTf_2_] were characterized by a lower degree of crystallinity (*X_c_*) (50.5–52.8%) as compared to PCLs obtained in [bmim][PF_6_] under the same experimental conditions (56.3–59.2%). The low *X_c_* values for PCLs is related to the high content of amorphous domains of cyclic macromolecules in the polymer structure that are formed as side products during eROP [25]. Their presence is the main factor determining the degradation rate of the polyester matrices. The amorphous domains in the PCL chain are mobile and have less packing, while the linear chains in the crystalline domains are rigid and take up less space. As a result, macrocycles show greater exposure of ester bonds to water during hydrolysis, so they degrade faster [26,27]. The content of cyclic oligomers can be controlled by selecting the appropriate reaction conditions. Analysis of the PCL structure determined by MALDI-ToF-MS and DSC showed that the highest content of macrocycles (35%) and the highest proportion of the amorphous phase (about 49.5%) was noted for PCL-1 synthesized in [bmim][NTf_2_] at 80 °C.

To evaluate the biocompatibility of the matrices, which is extremely important for medical devices like internal implants or urinary and venous catheters, we performed toxicity assays on the selected PCL samples synthesized in different ILs. For the first bioassay, the *umu*-test was used to evaluate the genotoxic potential of the tested materials. The genotoxic potential of the sample is presented as the induction ratio (IR). Results with IR ≥ 1.5 are considered genotoxic. Additionally, bacteria growth (G) is evaluated by a measurement of optical density to determine the toxicity of the tested samples.

All tested samples (Table 2) were not toxic for *Salmonella typhimurium* TA1535 (G > 0.5). The IR rises as a result of different types of DNA damage that induces the umuC gene in the SOS system linked in this bacterial strain to the synthesis of β-galactosidase. The IR ratio of both samples did not differ from the results obtained for negative or solvent control, therefore none of the tested PCLs matrices exhibited genotoxic activity (IR < 1.5). Similar negative results were evaluated with and without metabolic activation which disclaims the activity of tested samples as a procarcinogens in this bioassay.

In the next step, two in vitro cytotoxicity assays were performed using the BALB/c mouse 3T3 fibroblast cell line. The results revealed that in the NRU assay, BALB/c 3T3 cell viability was not decreased by any of the tested samples (Table 3).

**Figure 2:**
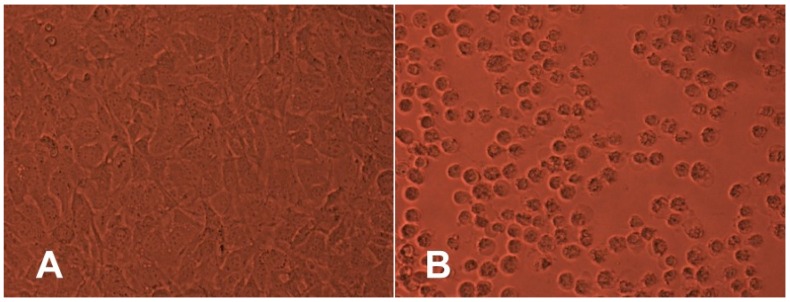
Sample PCL-1: (**A**) no detectable zone around or under specimen; (**B**) positive control zone with degenerated cells extending up to 0.7 cm around the specimen. Magnification × 200.

The percentage of viable cells did not differ significantly from the negative control. Moreover, the agar overlay assay also did not reveal any cytotoxic potential of any tested polyesters after 24 h of contact through the agar layer. The results show that there were no changes in the integrity of culture monolayer or general morphology of cells in comparison with the culture control (Figure 2). Moreover, there was no detectable zone around or under the specimen.

### 3.2. In Vitro Kinetics Release of CIP and TEMP from New Devices

**Figure 3:**
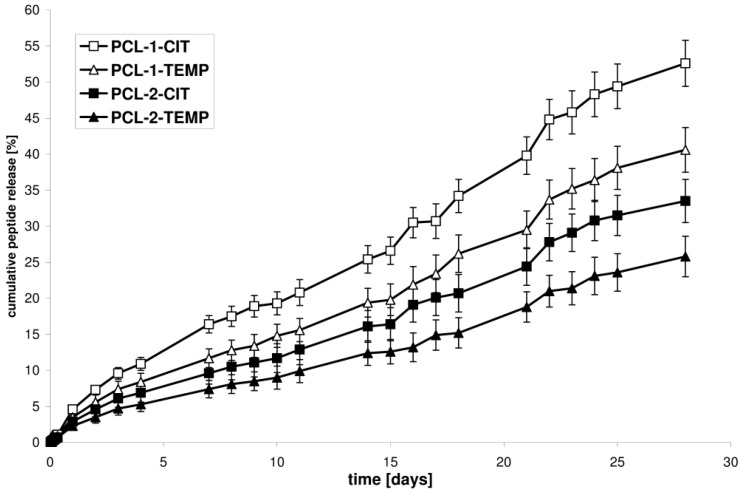
Cumulative release of peptides from PCL-1-CIT, PCL-1-TEMP, PCL-2-CIT, and PCL-2-TEMP over 28 days (each point represents mean ± SD of three points).

The kinetic release of CIP or TEMP from the selected synthesized matrices (PCL-1-CIT and PCL-1-TEMP, obtained from the PCL-1 matrix; PCL-2-CIT and PCL-2-TEMP, obtained from the PCL-2 matrix) was determined at pH 7.4 and 37 °C over 28 days (Figure 3). The ordinate of the plot was calculated based on the cumulative amount of peptide released [28].

It was found that the difference in the release rate observed for the PCL devices was attributed to the difference in the *X_c_* of the PCL matrices. The rate of in vitro peptide release increased as the *X_c_* of the matrices decreased. The percentage of CIT released after 28 days of incubation was about 52.6% for PCL-1-CIT (obtained from PCL-1, *X_c_* = 50.5%) and 33.5% for PCL-2-CIT (obtained from PCL-2, *X_c_* = 56.3%). Similarly, 40.6% of TEMP was released from PCL-1-TEMP (obtained from PCL-1, *X_c_* = 50.5%) and 25.8% from PCL-2-TEMP (obtained from PCL-2, *X_c_* = 56.3%). It was also found that CIT and TEMP were released in a rather regular and continuous way. Furthermore, CIT was released more quickly than TEMP. For instance, the amount of peptide released from PCL-1-CIT was about 16.4, 25.4, 39.8, and 52.6% after 7, 14, 21, and 28 days of incubation, respectively, whereas for PCL-1-TEMP these values were about 11.7, 19.4, 29.5, and 40.6% after 7, 14, 21, and 28 days of incubation, respectively. These differences were probably related to differences in the hydrophobic nature of the peptides. The amount of hydrophobic residue was 61 and 56% for TEMP and CIT, respectively. As a result, TEMP interact stronger with the hydrophobic polymeric matrix. Moreover, a high content of non-polar amino acids in the TEMPs structure (85%) provide to a poor solvation in water as compared to CIT (75% of the non-polar hydrophobic residues).

The release data points were subjected to zero-order and first-order kinetics and the Korsmeyer–Peppas model in order to evaluate the kinetics and mechanisms of peptide release from the obtained polyester matrices (Table 4).

As is commonly known, according to the Korsmeyer–Peppas model, for the diffusion-degradation–controlled peptide release system, the exponent value (*n*) varies between 0.45 and 0.89 (anomalous, non-Fickian). When *n* was close to 0.45, diffusion (Fickian diffusion) dominated in the process. When *n* was close to 0.89 (zero-order release), degradation controlled the release [22,23,24].

Our work shows that the obtained devices demonstrated a rather controlled release profile. High *R*^2^ values (from 0.9841 to 0.9877) were obtained for the near-zero-order kinetics model. The *R*^2^ values of the Korsmeyer–Peppas model were also high (from 0.9722 to 0.9797). The controlled drug release profiles were obtained with no significant burst release. This suggests that AMP release from the new PCLs matrices is a highly controlled process. Moreover, Table 4 shows that the exponent *n* in the Korsmeyer–Peppas model was approximately above 0.89. This is likely due to a rather degradation-controlled mechanism. The lower peptide release rate from PLA-2 than PLA-1 matrices is a logical consequence of the lower matrices’ crystallinity, because the degradation process is initiated in the amorphous phase [26].

### 3.3. Analysis of Matrix Surfaces After Degradation

**Figure 4:**
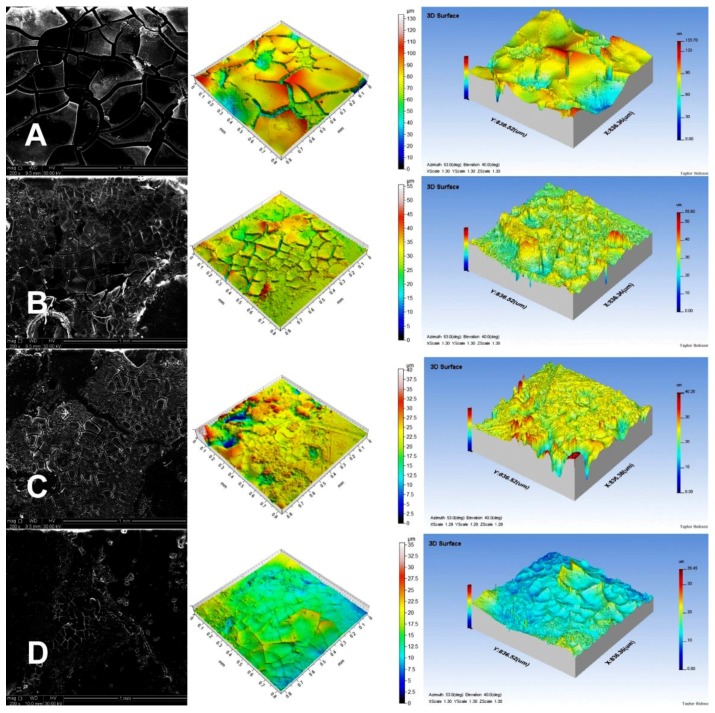
SEM micrographs and profilometer images showing the microstructure of PCL tablets after 28 days in PBS solution at 37 °C and pH 7.4: (**A**) PCL-1-CIT; (**B**) PCL-1-TEMP; (**C**) PCL-2-CIT; (**D**) PCL-2-TEMP.

Noncontact laser profilometry and SEM were used to determine the structural changes during the matrix degradation process. Figure 4 presents SEM micrographs and profilometer images of the microstructure of the PCL tablets after 28 d of incubation in PBS solution at 37 °C and pH 7.4.

We could easily see an increase in the depth of holes visible on the tablet surface in the following order: PCL-2-TEMP > PCL-2-CIT > PCL-1-TEMP > PCL-1-CIT. This occurrence confirms our previous assumption that PCL tablets synthesized in [bmim][NTf_2_] degraded faster than PCLs synthesized in [bmim][PF_6_] due to the lower crystallinity. 

Table 5 shows average roughness (*Ra*) of the tablets and mass loss after incubation in PBS. It was found that the difference in the release rate observed for the PCL devices was also attributed to the difference in *Ra* values of the PCL matrices. Profilometer analysis indicated that the highest *Ra* value (5.34) was obtained for PCL-1-CIT. For PCL-1-TEMP and PCL-2-CIT samples, the Ra values were 2.56 and 1.35, respectively. The lowest Ra value was obtained for the PCL-2-TEMP sample (about 0.32). Moreover, percentage mass loss was higher for PCL-1 than PCL-2 devices (54.2–22.8% and 16.4–7.9%, respectively), which is consistent with our previous report [20].

As it is commonly known, PCL is water-insoluble polymer. However, it is hydrolytically unstable and is degraded by hydrolysis of ester bonds. The process first occurs in the amorphous regions and is followed by a slower degradation in crystalline regions. Various studies have revealed that PCLs degradation is a result of several simultaneously occurring processes. These include water uptake, swelling, ester hydrolysis, diffusion of oligomers and degradation products, and local pH drop [29,30]. However, completely explanation of the mechanism which involves several factors is not yet possible.

### 3.4. Evaluation of Therapeutic Effect of New AMP Devices

**Figure 5:**
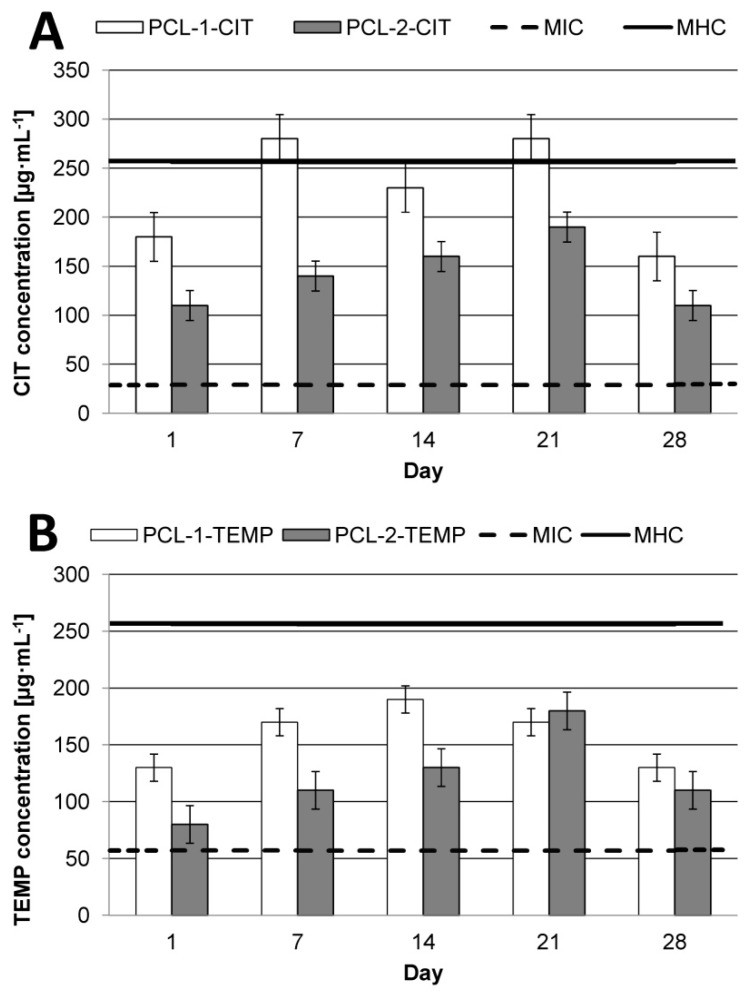
Peptide daily concentration after 1, 7, 14, 21, and 28 days of incubation in PBS solution at 37 °C and pH 7.4: (**A**) CIT; (**B**) TEMP. Geometric values of minimal inhibitory concentration (MIC) and minimal hemolytic concentration (MHC) were taken from the literature [13,31].

From the therapeutic point of view, it is very important to achieve therapeutic concentration of APIs in the target tissue. In the current work, we also investigated the daily release rate of AMPs after 1, 7, 14, 21, and 28 days (Figure 5) considering their reported MIC and MHC values [13,31]. We focused on the anti-infectious effect of AMPs in infections caused by major pathogens in surgical wound infections, *Staphylococcus aureus, Enterococcus faecalis*, *Escheria coli*, *Klebsiella pneumoniae*, and *Pseudomonas aeruginosa* [32].

CIT, due to its higher TI value (8.8), shows greater antimicrobial specificity as compared to TEMP (TI = 4.5). This indicates that CIT could be a promising antimicrobial compound for polymeric devices in wound infections. However, regardless the type of API, the type of matrix is a key factor for effective therapy. It was noted that PCL-2 was the best matrix for CIT. New PCL-2-CIT devices reach therapeutic concentration of AMPs (about 110–190 µg·mL^−1^) within 24 h and maintain it for the next 28 days. For TEMP, the most effective matrix appeared to be PCL-1, with peptide daily release of about 130–190 µg·mL^−1^ for 28 days.

For PCL-1, the peptide maximum daily concentration was dependent on the type of APIs. In the case of TEMP, maximum concentration was reached after 14 days of incubation in PBS at pH 7.4 at 37 °C. Interestingly, the maximum CIT daily release concentration was reached after 7 and 21 days (about 280 µg·mL^−1^), and this value exceeded the MHC values (about 256 µg·mL^−1^ [31]). For PCL-2, the highest daily concentration of both peptides was noted after 21 days.

## 4. Conclusions

In the present work, new nontoxic polymeric matrices for controlled CIT and TEMP release were obtained. PCLs with different crystallinity were successfully synthesized by eROP in two different ILs, [bmim][NTf_2_] and [bmim][PF_6_]. The in vitro release study demonstrated that CIT and TEMP release rates were strongly dependent on the crystallinity of the polymeric matrices. However, peptide characteristics were also important. CIT, a less hydrophobic peptide, was released more quickly than the more hydrophobic TEMP. We found that the peptides were released with high control, according to the degradation mechanism with near-zero-order kinetics. The peptide “burst release” was not observed during the degradation process. New devices reached a therapeutic concentration of AMPs within 24 h and maintained it for 28 days. From a broader perspective, this study suggests that synthesized polyester matrices may contribute to a potential application as medium- and long-term controlled CIT or TEMP delivery systems, offering fast antibacterial effects on local infections in implantology.

## Figures and Tables

**Table 1:** Characterization of poly(ε-caprolactone) (PCL) matrices synthesized in the presence of *Candida antarctica* (CALB) in ionic liquids (ILs).

No.	Solvent	Temp. [°C]	*M*_n_^a^ [Da]	*M*_w_/*M*_n_^b^	Yield [%]	Conv. ^c^ [%]	T_c_^d^ [°C]	T_m_^d^ [°C]	∆*H_f_* [J·g^−1^]	*X_c_* [%]	T_deg_ [°C]	MC ^e^ [%]
**PCL-1**	[bmim][NTf_2_]	80	6100	1.8	78	95	34.7	53.1	70.4	50.5	405.4	35
**PCL-2**	[bmim][PF_6_]	80	5700	1.5	87	92	28.5	50.6	78.4	56.3	410.3	19
**PCL-3**	[bmim][NTf_2_]	60	3600	2.0	88	90	32.1	50.4	73.7	52.8	406.9	23
**PCL-4**	[bmim][PF_6_]	60	2100	1.4	85	94	30.4	47.3	82.7	59.2	411.7	0
**PCL-5**	–	80	1000	2.4	61	73	31.6	49.1	75.7	54.2	407.9	0
**PCL-6**	–	60	2700	1.2	78	87	32.5	50.4	84.6	60.1	414.5	0

^a^ Determined by SEC using the correction coefficient *M*_n(SEC)_ = 0.56·*M*_n(SEC raw data)_; ^b^ determined by SEC; ^c^ determined by ^1^H NMR [20]; ^d^ onset temperature; ^e^ determined by MALDI-ToF-MS.

**Table 2:** The *umu*-test results for the highest concentrations of tested extracts (0.67 mg·mL^−1^).

Sample	−S9 ^a^		+S9 ^b^	
	G ± SD	IR ± SD	G ± SD	IR ± SD
**PCL-1**	0.90 ± 0.08	0.97 ± 0.14	0.87 ± 0.03	0.94 ± 0.07
**PCL-2**	0.92 ± 0.05	1.02 ± 0.09	0.88 ± 0.01	0.90 ± 0.08
**Solvent control**	1.01 ± 0.09	0.94 ± 0.15	0.88 ± 0.01	1.03 ± 0.06
**Negative control**	1.00 ± 0.05	1.00 ± 0.01	1.00 ± 0.05	1.00 ± 0.08

^a^ version without metabolic activation; ^b^ version with metabolic activation.

**Table 3:** Results of the NRU test for the highest concentrations of tested extracts (0.5 mg·mL^−1^).

Sample	Cell Viability ± SD (%)
**PCL-1**	96 ± 2
**PCL-2**	95 ± 1
**Solvent control**	100 ± 5

**Table 4:** Analysis data of peptide release from polymeric matrices.

No.	Zero-Order Model	First-Order Model	Korsmeyer–Peppas Model	
	*R*^2^	*R*^2^	*R*^2^	*n*
**PCL-1-CIT**	0.9877	0.9574	0.9797	>0.89
**PCL-1-TEMP**	0.9853	0.9603	0.9742	>0.89
**PCL- 2-CIT**	0.9855	0.9644	0.9732	>0.89
**PCL-2-TEMP**	0.9841	0.9656	0.9722	>0.89

**Table 5:** Parameters of surface roughness and mass loss after 28 days in PBS solution at 37 °C and pH 7.4.

No.	Ra ± SD	Mass Loss ± SD (%)
**PCL-1-CIT**	5.34 ± 1.60	54.2 ± 2.2
**PCL-1-TEMP**	2.56 ± 0.74	22.8 ± 1.7
**PCL-2-CIT**	1.35 ± 0.75	16.4 ± 0.9
**PCL-2-TEMP**	0.32 ± 0.10	7.9 ± 0.5
